# The School Climate and Academic Mindset Inventory (SCAMI): Confirmatory Factor Analysis and Invariance Across Demographic Groups

**DOI:** 10.3389/fpsyg.2020.02061

**Published:** 2020-08-14

**Authors:** Christopher A. Kearney, Ricardo Sanmartín, Carolina Gonzálvez

**Affiliations:** ^1^Department of Psychology, University of Nevada, Las Vegas, Las Vegas, NV, United States; ^2^Department of Developmental Psychology and Teaching, University of Alicante, Alicante, Spain

**Keywords:** school climate, school climate and academic mindset inventory, confirmatory factor analysis, academic mindset, social emotional learning

## Abstract

School climate is a multidimensional construct of the quality of a student’s academic environment, often subsuming dimensions such as safety, instructional practices, social relationships, school facilities, and school connectedness. Positive school climate has beneficial effects on a wide range of adjustment variables in youth, including academic achievement, mental health, school attendance and graduation, and school-based behavior. Studies regarding school climate assessment have burgeoned in recent years but remain marked by limited sample sizes, narrow developmental levels, restricted items, unclear psychometric strength across multiple demographic groups, and lack of integration with key student education contextual factors such as academic mindset and social emotional learning. The present study evaluated a comprehensive measure of aspects of school climate, academic mindset, and social emotional learning in a sample of 105,379 diverse students (*M*_age_ = 13.47 years; *SD* = 2.47). The 9-factor structure of the School Climate and Academic Mindset Inventory was supported via confirmatory factor analysis. A trimmed model displayed adequate goodness-of-fit for males and females, younger age groups, and European American, biracial/multiracial, Hispanic, Native American, and Native Hawaiian/Pacific Islander students. The trimmed model was slightly less strong for older age groups as well as for African American and Asian American students. The scale may be useful for assessing school climate interventions, longitudinal climate patterns, and school-based algorithms of future performance, though additional validation of the scale remains necessary.

## Introduction

School climate is a multidimensional construct of the “quality and character of school life” that reflects “norms, goals, values, interpersonal relationships, teaching and learning practices, and organizational structures” in a youth-based academic environment (Cohen et al., 2009; Thapa and Cohen, 2017, pp. 303–304). Key dimensions of school climate include safety, instructional practices, social relationships, school facilities, and school connectedness (Zullig et al., 2010). Other, related dimensions of school climate include community, equity, institutional or academic environment, leadership, and shared beliefs (Uline et al., 2010; Wang and Degol, 2016).

Dimensions of school climate have been further explicated in the literature. Safety refers to actual and perceived degree of relational aggression as well as respect for others, supportive environments, and clear rules and norms (Goldstein et al., 2008; Capp et al., 2020). Teaching and learning practices refer to quality of instruction, social emotional learning, and support for academic achievement (Gage et al., 2016). Social relationships refer to parent involvement, supportive relationships with important others such as peers and teachers at school, and respect for diversity (Bradshaw et al., 2014b; Gage et al., 2014). School facilities refer to the condition of the physical environment of an academic setting as well as availability of resources and supplies (Gislason, 2010). School connectedness refers to student affective attachment to the academic community, commitment to social and academic goals, and involvement in social and academic activities (García-Moya et al., 2019). Several of these dimensions overlap in definition and scope, most notably degree of support from the academic environment (Thapa et al., 2013; McMillan et al., 2017).

School climate has been linked to improved child and adolescent socioemotional and academic adjustment in many ways. Positive school climate has been associated with enhanced academic achievement, particularly among students from lower socioeconomic backgrounds (Berkowitz et al., 2017; Maxwell et al., 2017). In addition, positive or authoritative school climate is associated with various mental health benefits that include increased self-esteem, psychosocial wellbeing, and perceived quality of life as well as less depression, suicidal thoughts and behavior, substance use, and psychiatric problems (Thapa et al., 2013; Cornell and Huang, 2016; Aldridge and McChesney, 2018; Zullig et al., 2018). Positive school climate is also linked to academic behavioral outcomes such as less absenteeism, school dropout, suspensions, disciplinary referrals, and bullying and other violent school-based incidents (Kutsyuruba et al., 2015; Hendron and Kearney, 2016; Jia et al., 2016; Reaves et al., 2018). More broadly, positive school climate relates to enhanced teacher satisfaction, retention, efficacy, and productivity as well as less burnout (Berkowitz et al., 2017).

School climate enhancement has thus become a central aspect of broad-based school improvement strategies. Such efforts prominently include, for example, Positive Behavioral Interventions and Supports (PBIS), a systemic intervention to promote a positive school environment that includes appropriate student and teacher behavior (Sugai and Horner, 2002). Key aspects of PBIS include clearly articulated behavioral expectations, student incentives, positive student-teacher interactions, effective classroom management, and empirically based decision making (Bradshaw et al., 2014a). Efforts to improve school climate are typically integrated into multi-tiered systems of support models that focus in part on primary prevention practices to promote adaptive behavior and to deter maladaptive behavior (Lewis et al., 2017). PBIS implemented with fidelity relates to improvements in academic performance, bullying, office discipline referrals, social emotional competencies, and student suspensions and expulsions (Bradshaw et al., 2010; Horner and Sugai, 2015; Freeman et al., 2016).

The importance of school climate to child and adolescent adjustment as well as to overall academic and behavioral outcomes mandates the need for comprehensive and psychometrically strong assessment measures for this construct, particularly for schools (Lindstrom Johnson et al., 2019). A full review of school climate assessments is beyond the scope of this article (for reviews, see Ramelow et al., 2015; Olsen et al., 2018). These measures focus primarily on various dimensions of school climate described earlier in addition to ancillary variables such as substance use, school-community relations, and school identification (e.g., Lee et al., 2017). Substantial variability exists across the measures with respect to school climate domains and how these domains are labeled (Shukla et al., 2019).

Despite the availability of instruments to measure school climate, researchers have noted shortcomings with respect to many of these measures and their associated psychometric properties (Ramelow et al., 2015; Olsen et al., 2018). First, many measures in this area are not particularly comprehensive, instead focusing on limited numbers of constructs and items related only to safety, student-teacher relationships, and connectedness (Berkowitz et al., 2017). Second, psychometric support for most school climate assessments is limited and is often based on restricted sample sizes and developmental levels (Wang and Degol, 2016). Third, important student-based contextual variables related to school climate are often neglected in these measurements (Konold, 2018). Examples include key academic mindset and social emotional learning competencies utilized by students in their academic endeavors (Rattan et al., 2015; Lawson et al., 2019). Theories of optimal school performance in children often integrate student-based contextual factors with school climate (Lee and Shute, 2010; Rudasill et al., 2018).

Academic mindsets refer to “beliefs, attitudes, or ways of perceiving oneself in relation to learning and intellectual work that support academic performance” (Farrington et al., 2012, p. 28). Such intrinsic motivational or non-cognitive factors, including academic tenacity or self-efficacy, relate closely to improved academic achievement across many demographic groups (Yeager and Dweck, 2012; Dweck et al., 2014). Organizational learning variables that can include a positive school climate correlate significantly with a growth mindset culture among students (Hanson et al., 2016). Mechanisms for this relationship may include enhanced student sense of contextual fairness as well as solidarity or belongingness at school, incentives for academic performance, trust in authorities, and transmission of mindset beliefs by teachers (Thomas et al., 2019).

Social emotional learning competencies, sometimes integrated with the teaching and learning practices domain of school climate, refer broadly to skills regarding self-awareness, self-management, social awareness, relationships, and responsible decision-making (Durlak et al., 2015). Social emotional learning practices can help produce adaptive academic mindsets via (1) explicit instruction in interpersonal, emotional, and cognitive skills, (2) ample school-based opportunities to use these skills, and (3) effective classroom management and discipline approaches (Darling-Hammond and Cook-Harvey, 2018). Efforts to enhance school climate often include social-emotional learning practices to promote safe contexts to develop these skills and to boost student engagement (Corcoran et al., 2018).

The aim of the present study was to evaluate a comprehensive measure of aspects of school climate, academic mindset, and social emotional learning in a very large sample of diverse students. The study was designed in part to help address drawbacks associated with extant measures in this area such as restricted comprehensiveness and sample size, limited psychometric strength across multiple demographic groups, and lack of integration with key contextual factors related to student learning. Specifically, the present study examined the adjustment and reliability of a districtwide 9-factor, 66-item measure of school climate and academic mindset (School Climate and Academic Mindset Inventory; SCAMI) among tens of thousands of elementary, middle, and high school students. The primary hypothesis was that the original 9-factor model representing these constructs (i.e., parent involvement and support, academic mindset, social emotional learning, safety, physical safety, bullying, physical environment and resources, respect for diversity, and perceptions of school performance) would be supported via confirmatory factor analysis. In addition, factorial invariance across multiple demographic groups was expected. Model trimming was implemented as needed.

## Materials and Methods

### Participants

Participants included 4th–12th grade students (*n* = 105,379) in a large urban school district in the United States. Participants were slightly more female (50.1%), aged 9–21 years (*M* = 13.47; *SD* = 2.47), and Hispanic/Latino (45.5%), European American (27.7%), African American (10.3%), Asian (8.2%), biracial/multiracial (6.3%), Native Hawaiian/Pacific Islander (1.6%), and American Indian/Alaska Native (0.4%). Distribution by age group follows: 9–11 years (39,860), 12–14 years (27,116), 15–16 years (21,590), 17–18 years (16,228), and >18 years (585). Participants voluntarily completed an online survey of the measure described next during the spring semester of the 2016–2017 academic year. The survey was posted publicly by the school district and via social media, but the largest contingent of students completed the measure at their school. Each school was encouraged to survey 75% of their population, but the overall response rate is unknown.

### Measure and Data Analyses

Original subscales and associated items are in Table 1. Subscales (*n* = 9) included Parent Involvement and Support, Academic Mindset, Social Emotional Learning, Safety, Physical Safety, Bullying, Physical Environment and Resources, Respect for Diversity, and Perceptions of School Performance. Items were derived by the school district via original development, a state department of education, and the University of Chicago Consortium on School Research (academic mindset questions). Items are scored in variable fashion (Table 1).

**TABLE 1:** School Climate and Academic Mindset Inventory items (original model).

**Item**	**Key**	**Subscale**
My parents feel welcome to come to my school	1 = *strongly disagree*	Parent Involvement and Support
This school involves parents in most school events or activities	2 = *disagree*	
My parents know what goes on inside my school	3 = *agree*	
	4 = *strongly agree*	
My intelligence is something that I can’t change very much	1 = *not at all true*	Academic Mindset
Challenging myself won’t make me any smarter	2 = *a little true*	
There are some things I am not capable of learning	3 = *somewhat true*	
If I am not naturally smart in a subject, I will never do well in it	4 = *mostly true*	
I don’t participate in discussions because I am afraid people might think I am foolish	5 = *completely true*	
I would rather do easy work that I can do well than challenging work where I might learn more*		
I don’t ask questions in class because people might think my questions are not smart		
I stop doing work if I feel like I can’t do it well*		
I only volunteer to answer a question if I am sure my answer is right		
Do the readings or other assigned work to prepare for class?	1 = *never*	
Turn in assignments on the due date?	2 = *once in a while*	
Actively participate in class?	3 = *about half the time*y	
Have all of your class materials with you?	4 = *most of the time*	
Do more than what is expected of you?	5 = *always*	
Knowing what my strengths are*	1 = *very difficult*	Social Emotional Learning
Knowing ways I calm myself down*	2 = *difficult*	
Knowing the emotions I feel*	3 = *easy*	
Knowing when my feelings are making it hard for me to focus*	4 = *very easy*	
Knowing what people may be feeling by the look on their face*		
Learning from people with different opinions than me*		
Knowing when someone needs help*		
Getting through something even when I feel frustrated		
Being patient even when I am really excited		
Finishing tasks even if they are hard for me		
Setting goals for myself		
Doing my schoolwork even when I do not feel like it		
Being prepared for tests		
Getting along with my classmates*		
Respecting a classmate’s opinions during a disagreement*		
Thinking about what might happen before making a decision*		
Knowing what is right or wrong*		
This school is safe	1 = *strongly disagree*	Safety
Students feel safe in this school	2 = *disagree*	
This school has safety procedures that work	3 = *agree*	
Students know what to do if there is an emergency during school	4 = *strongly agree*	
This school encourages me to have healthy habits (ex., physical activity or nutrition)		
I sometimes stay home because I don’t feel safe at this school	1 = *strongly disagree*	Physical Safety
Students at this school threaten to hurt other students	2 = *disagree*	
Students at this school damage or destroy other students’ property	3 = *agree*	
	4 = *strongly agree*	
This school does a good job to prevent bullying	1 = *strongly disagree*	Bullying
Students in this school are teased about their clothing or physical appearance	2 = *disagree*	
Bullying is a problem at this school	3 = *agree*	
Cyberbullying is a problem at this school	4 = *strongly agree*	
Students in this school are teased or put down because of their race or ethnicity		
In my experience, at this school everything works or gets fixed quickly	1 = *strongly disagree*	Physical Environment and Resources
This school is clean	2 = *disagree*	
The heating and air conditioning work well at this school	3 = *agree*	
The technology (computers, iPads, mobile devices, etc.) works well at this school	4 = *strongly agree*	
The equipment and facilities at this school work well		
School staff treat students with respect, regardless of differences like race, ethnicity, gender, or disability	1 = *strongly disagree*	Respect for Diversity
	2 = *disagree*	
This school encourages an appreciation of student diversity and respect for each other	3 = *agree*	
School staff encourages all students to take challenging courses no matter their race, ethnicity, gender, or disability	4 = *strongly agree*	
Student treat other students with respect, regardless of differences like race, ethnicity, gender, or disability		
My school encourages me to be courteous and respectful toward others		
At my school, my teachers tell me how I am doing in my classes	1 = *strongly disagree*	Perceptions of School Performance
This school promotes academic success for all students	2 = *disagree*	
I am learning with technology such as computers, mobile devices and the Internet at this school	3 = *agree*	
I like my school*	4 = *strongly agree*	
I am getting a good education at this school		
Help is available at this school if I have trouble with my schoolwork		
Teachers understand my problems		
My teachers care about me*		
If I am absent, there is a teacher or some other adult at school that will notice my absence		

*Removed items in trimmed model.

The subscales had different Likert-type scales, so raw data were lineally transformed to obtain scores on a 0–100 scale (see Vilagut et al., 2005). Items were reversed-scored as needed. Confirmatory factor analyses (CFA) were then performed to analyze models that could be adjusted to the data: 0-factor model, 1-factor model, original 9-factor model (with correlated and uncorrelated factors), a trimmed 9-factor model (with correlated and uncorrelated factors), and a trimmed 9-factor model with a second-order factor (with correlated and uncorrelated factors). A trimmed 9-factor model was obtained by analyzing item saturation in each of the factors, keeping the theoretical meaning of the items inside the respective factors and the modification indices (Leung et al., 2013; Gonzálvez et al., 2016; Gómez-Núñez et al., 2020). Mardia’s coefficient of the total sample was above the 5 points established as multivariate normality in the data (1,122.76), so the Robust Maximum Likelihood (RML) and Satorra-Bentler χ^2^ scaled (S-Bχ^2^) were applied (Bentler, 2005). To identify extreme cases with respect to multivariate kurtosis, the method that was employed was the analysis of the five cases automatically provided by EQS that contribute most to the normalized multivariate kurtosis estimate. The criterion used was the comparison between the estimate presented for one case relative to the estimate of the other four cases. This method was implemented until the five estimates of the final sample were included in the same range of values and none was distinctively different from the others (Byrne, 2008).

Four goodness-of-fit indices were examined for the models: robust root mean square error of approximation (R-RMSEA), standardized root mean square residual (SRMR), robust comparative fit index (R-CFI), and Tucker Lewis index (TLI). Acceptable goodness-of-fit in this study was defined as rounded SRMR and R-RMSEA values of <0.08 and R-CFI and TLI values of 0.90+ (Hu and Bentler, 1999; Brown, 2006). In addition, the upper end of the R-RMSEA 90% confidence interval should be <0.10 (Kline, 2005). Subscale reliability of the best-fitting model was evaluated via Cronbach’s alpha and Omega coefficients (McDonald, 1999).

Multigroup confirmatory factor analyses were performed to test factorial invariance of the chosen model (configural, measurement, and structural invariance) across gender, age group (9–11, 12–14, 15–16, 17–18, 18+ years) and ethnic group (Hispanic/Latino, European American, African American, Asian, Biracial/Multiracial, Native Hawaiian/Pacific Islander, and American Indian/Alaska Native). Mardia’s coefficients were elevated, so the S-Bχ^2^ and the robust indices mentioned above were used to include certain groups in the invariance analyses and determine the adequacy of the nested models that are part of the invariance analyses. An invariance criterion (ΔR-CFI > −0.01) was also utilized to accept nested models (Byrne, 2008). Statistical analyses were calculated using SPSS, Amos 23, and EQS 6.1.

## Results

### Confirmatory Factor Analyses and Reliability

Results from the CFAs are in Table 3. The 0-factor, 1-factor, and original 9-factor model with and without correlated factors did not meet criteria for adequate goodness-of-fit. Model trimming then consisted of removing 15 items whose factor loadings were <0.40 (noted in Table 1). These items included 2 from academic mindset, 11 from social emotional learning, and 2 from perceptions of school environment. Errors were correlated to improve model fit. No items were removed from the other subscales, and no items were moved from one subscale to another (Table 2). Removal of these 15 items produced a trimmed 9-factor 51-item model for the SCAMI with and without correlated factors that was not supported by all goodness-of-fit indices. A new trimmed 9-factor model dividing factor 2 (academic mindset) into a second-order factor was proposed (Table 3). The trimmed 9-factor model with a second-order factor with correlated factors met criteria for adequate goodness-of-fit (R-RMSEA = 0.034, 90% confidence interval: 0.34–0.34; SRMR = 0.047; R-CFI = 0.908; TLI = 0.901).

**TABLE 2:** School Climate and Academic Mindset Inventory items (trimmed model).

**Item**	**Key**	**Subscale**
My parents feel welcome to come to my school	1 = *strongly disagree*	Parent Involvement and Support
This school involves parents in most school events or activities	2 = *disagree*	
My parents know what goes on inside my school	3 = *agree*	
	4 = *strongly agree*	
My intelligence is something that I can’t change very much	1 = *not at all true*	Academic Mindset
Challenging myself won’t make me any smarter	2 = *a little true*	
There are some things I am not capable of learning	3 = *somewhat true*	
If I am not naturally smart in a subject, I will never do well in it	4 = *mostly true*	
I don’t participate in discussions because I am afraid people might think I am foolish	5 = *completely true*	
I don’t ask questions in class because people might think my questions are not smart		
I only volunteer to answer a question if I am sure my answer is right		
Do the readings or other assigned work to prepare for class?	1 = *never*	
Turn in assignments on the due date?	2 = *once in a while*	
Actively participate in class?	3 = *about half the time*	
Have all of your class materials with you?	4 = *most of the time*	
Do more than what is expected of you?	5 = *always*	
Getting through something even when I feel frustrated	1 = *very difficult*	Social Emotional Learning
Being patient even when I am really excited	2 = *difficult*	
Finishing tasks even if they are hard for me	3 = *easy*	
Setting goals for myself	4 = *very easy*	
Doing my schoolwork even when I do not feel like it		
Being prepared for tests		
This school is safe	1 = *strongly disagree*	Safety
Students feel safe in this school	2 = *disagree*	
This school has safety procedures that work	3 = *agree*	
Students know what to do if there is an emergency during school	4 = *strongly agree*	
This school encourages me to have healthy habits (ex., physical activity or nutrition)		
I sometimes stay home because I don’t feel safe at this school	1 = *strongly disagree*	Physical Safety
Students at this school threaten to hurt other students	2 = *disagree*	
Students at this school damage or destroy other students’ property	3 = *agree*	
	4 = *strongly agree*	
This school does a good job to prevent bullying	1 = *strongly disagree*	Bullying
Students in this school are teased about their clothing or physical appearance	2 = *disagree*	
Bullying is a problem at this school	3 = *agree*	
Cyberbullying is a problem at this school	4 = *strongly agree*	
Students in this school are teased or put down because of their race or ethnicity		
In my experience, at this school everything works or gets fixed quickly	1 = *strongly disagree*	Physical Environment and Resources
This school is clean	2 = *disagree*	
The heating and air conditioning work well at this school	3 = *agree*	
The technology (computers, iPads, mobile devices, etc.) works well at this school	4 = *strongly agree*	
The equipment and facilities at this school work well		
School staff treat students with respect, regardless of differences like race, ethnicity, gender, or disability	1 = *strongly disagree*	Respect for Diversity
	2 = *disagree*	
This school encourages an appreciation of student diversity and respect for each other	3 = *agree*	
School staff encourages all students to take challenging courses no matter their race, ethnicity, gender, or disability	4 = *strongly agree*	
Student treat other students with respect, regardless of differences like race, ethnicity, gender, or disability		
My school encourages me to be courteous and respectful toward others		
At my school, my teachers tell me how I am doing in my classes	1 = *strongly disagree*	Perceptions of School Performance
This school promotes academic success for all students	2 = *disagree*	
I am learning with technology such as computers, mobile devices and the Internet at this school	3 = *agree*	
I am getting a good education at this school	4 = *strongly agree*	
Help is available at this school if I have trouble with my schoolwork		
Teachers understand my problems		
If I am absent, there is a teacher or some other adult at school that will notice my absence		

**TABLE 3:** Goodness-of-fit indices for proposed models.

	**S-Bχ^2^**	**df**	**R-RMSEA 90% CI**	**SRMR**	**R-CFI**	**TLI**
0-factor model	2051321.19	2145	0.095 [0.095,0.095]	0.225	0.000	0.000
1-factor model	841569.57	2079	0.062 [0.062,0.062]	0.079	0.590	0.577
Original 9-factor model with non-correlated factors	706350.10	2079	0.057 [0.057,0.057]	0.193	0.656	0.645
Original 9-factor model with correlated factors	339922.74	2043	0.040 [0.040,0.040]	0.056	0.835	0.827
Trimmed 9-factor model with non-correlated factors	585501.18	1221	0.067 [0.067,0.068]	0.214	0.634	0.617
Trimmed 9-factor model with correlated factors	221087.08	1185	0.042 [0.042,0.042]	0.058	0.862	0.852
Trimmed 9-factor model with one second-order factor with non-correlated factors	515599.04	1218	0.063 [0.063,0.063]	0.212	0.678	0.662
Trimmed 9-factor model with one second-order factor with correlated factors	148025.13	1182	0.034 [0.034,0.034]	0.047	0.908	0.901

**p* < 0.001 for S-Bχ^2^ in all cases. S-Bχ^2^ = Satorra-Bentler scaled χ^2^; df = degrees of freedom; R-RMSEA = robust root mean square error of approximation; CI = confidence interval; SRMR = standardized root mean square residual; R-CFI = robust comparative fit index; TLI = Tucker Lewis Index.*

Factor loadings of the trimmed 9-factor model with one second-order factor with correlated factors ranged from 0.43–0.83. Internal consistency coefficients (Cronbach’s alpha/Omega/Composite reliability/Average Variance Extracted) were calculated for each subscale: Parent involvement and Support (0.61/0.61/0.61/0.35), Academic Mindset (0.75/0.87/0.87/0.37), Social Emotional Learning (0.75/0.76/0.76/0.35), Safety (0.84/0.83/0.83/0.50), Physical Safety (0.72/0.74/0.74/0.50), Bullying (0.81/0.80/0.80/0.45), Physical Environment and Resources (0.78/0.78/0.78/0.41), Respect for Diversity (0.79/0.80/0.80/0.45), and Perceptions of School Performance (0.83/0.84/0.84/0.42).

### Factorial Invariance Across Demographic Groups

Factorial invariance of the trimmed 9-factor model with one second-order factor was examined across several demographic groups. A baseline model (Model 0) without constraints was initially established; factor loadings of first order (Model 1) and second order (Model 2) models were then imposed to obtain metric invariance. In addition, intercept constraints were added to Model 2 and the strong or scalar invariance (Model 3) was obtained. Factor loadings (first and second order), intercepts, and variances and covariances of errors were constrained to test strict invariance (Model 4). Finally, covariances of the factors were constrained in Model 2 to test structural invariance (Model 5).

Regarding invariance across gender, the trimmed model displayed adequate goodness-of-fit criteria for males and females (TLI and R-CFI > 0.90; R-RMSEA < 0.05; SRMR < 0.08; ΔR-CFI values > −0.01) (Table 4). Measurement and structural invariance were thus confirmed (adequate goodness-of-fit indexes values and ΔR-CFI > −0.01 for all tested models). Regarding invariance across age, the trimmed model displayed adequate goodness-of-fit criteria for younger age groups: 9–11 years and 12–14 years (TLI and R-CFI > 0.90; R-RMSEA < 0.05; SRMR < 0.08) (Table 3) but less so for older age groups: 15–16 years (R-RMSEA = 0.037, 90% confidence interval: 0.37–0.37; SRMR = 0.049; R-CFI = 0.894; TLI = 0.886); 17–18 years (R-RMSEA = 0.040, 90% confidence interval: 0.39–0.40; SRMR = 0.054; R-CFI = 0.884; TLI = 0.875); 18+ years (R-RMSEA = 0.039, 90% confidence interval: 0.37–0.42; SRMR = 0.073; R-CFI = 0.891; TLI = 0.883). Factorial invariance was thus tested for the younger age groups; adequate goodness-of-fit criteria were met and all ΔR-CFI > −0.01 (Table 5). Measurement and structural invariance were thus confirmed.

**TABLE 4:** Goodness-of-fit indices for invariance of the trimmed model across gender.

	***χ*^2^**	**S-Bχ^2^**	**df**	**TLI**	**R-CFI**	**R-RMSEA**	**SRMR**	****Δ**R-CFI**
Male	91026.93	72675.50	1182	0.900	0.904	0.034 [0.034, 0.034]	0.049	
Female	97394.50	78792.53	1182	0.903	0.910	0.035 [0.035, 0.035]	0.046	
Model 0	188421.42	151427.68	2364	0.900	0.907	0.024 [0.024, 0.025]	0.048	
Model 1	189471.73	152657.94	2412	0.901	0.906	0.024 [0.024, 0.024]	0.049	−0.001
Model 2	189641.89	152816.94	2415	0.901	0.906	0.024 [0.024, 0.024]	0.049	0.000
Model 3	199691.90	162146.73	2466	0.900	0.907	0.025 [0.025, 0.025]	0.049	0.001
Model 4	211280.97	170001.21	2523	0.900	0.902	0.025 [0.025, 0.025]	0.051	−0.005
Model 5	200555.12	162554.53	2502	0.901	0.906	0.025 [0.025, 0.025]	0.049	−0.001

*Model 0 = free model; Model 1 = Model 0 with factor loadings of first-order; Model 2 = Model 1 with factor loadings of second order; Model 3 = Model 2 with intercepts; Model 4 = Model 3 with error variances and covariances; Model 5 = Model 3 with factor covariances; S-Bχ^2^ = Satorra-Bentler χ^2^ scaled; df = degrees of freedom; TLI = the Tucker-Lewis Index; R-CFI = robust comparative fit index; R-RMSEA = robust root mean square error of approximation; SRMR = standardized root mean square residual; ΔR-CFI = robust comparative fit index difference test.*

**TABLE 5:** Goodness-of-fit indices for invariance of the trimmed model across age.

	***χ*^2^**	**S-Bχ^2^**	**df**	**TLI**	**R-CFI**	**R-RMSEA**	**SRMR**	****Δ**R-CFI**
9–11 years	52629.06	42181.28	1182	0.910	0.917	0.030 [0.029, 0.030]	0.040	
12–14 years	49769.53	40101.60	1182	0.900	0.904	0.035 [0.035, 0.035]	0.046	
Model 0	102398.60	82288.41	2364	0.904	0.911	0.022 [0.022, 0.023]	0.044	
Model 1	103865.77	83586.59	2412	0.905	0.910	0.022 [0.022, 0.023]	0.051	−0.001
Model 2	104034.29	83732.25	2415	0.905	0.910	0.022 [0.022, 0.023]	0.051	0.000
Model 3	120179.21	97916.29	2466	0.900	0.902	0.024 [0.024, 0.024]	0.053	−0.008
Model 4	128118.79	1009211.70	2523	0.904	0.910	0.025 [0.025, 0.025]	0.053	0.008
Model 5	121071.88	98409.56	2502	0.900	0.902	0.024 [0.024, 0.024]	0.051	0.000

*Model 0 = free model; Model 1 = Model 0 with factor loadings of first-order; Model 2 = Model 1 with factor loadings of second order; Model 3 = Model 2 with intercepts; Model 4 = Model 3 with error variances and covariances; Model 5 = Model 3 with factor covariances; S-Bχ^2^ = Satorra-Bentler χ^2^ scaled; df = degrees of freedom; TLI = the Tucker-Lewis Index; R-CFI = robust comparative fit index; R-RMSEA = robust root mean square error of approximation; SRMR = standardized root mean square residual; ΔR-CFI = robust comparative fit index difference test.*

Regarding invariance across ethnic group, the trimmed model displayed adequate goodness-of-fit criteria for European American, Biracial/Multiracial, Native Hawaiian/Pacific Islander, Hispanic, and American Indian/Alaska Native students (TLI and R-CFI > 0.90; R-RMSEA < 0.05; SRMR < 0.08) (Table 6). Goodness-of-fit was less strong for African-American (R-RMSEA = 0.034, 90% confidence interval: 0.34–0.35; SRMR = 0.052; R-CFI = 0.902; TLI = 0.894) and Asian American (R-RMSEA = 0.036, 90% confidence interval: 0.35–0.36; SRMR = 0.047; R-CFI = 0.900; TLI = 0.888) students. Metric invariance was not confirmed (TLI < 0.90 and SRMR > 0.08 for Model 2; factorial invariance analyses were thus halted).

**TABLE 6:** Goodness-of-fit indices for invariance of the trimmed model across ethnic group.

	***χ*^2^**	**S-Bχ^2^**	**df**	**TLI**	**R-CFI**	**R-RMSEA**	**SRMR**	****Δ**R-CFI**
European American	56198.61	45105.27	1182	0.902	0.909	0.036 [0.035, 0.036]	0.045	
Biracial/Multiracial	13515.08	10854.09	1182	0.900	0.908	0.035 [0.035, 0.036]	0.047	
Native Hawaiian/Pacific Islander	4401.75	3517.27	1182	0.900	0.907	0.034 [0.033, 0.035]	0.054	
Hispanic	80159.60	64407.09	1182	0.902	0.909	0.033 [0.033, 0.034]	0.048	
American Indian/Alaska Native	2182.76	1769.76	1182	0.911	0.917	0.034 [0.030, 0.037]	0.065	
Model 0	156457.79	125759.75	5910	0.903	0.910	0.015 [0.015, 0.0015]	0.052	
Model 1	157851.85	127360.183	6102	0.905	0.909	0.015 [0.015, 0.0015]	0.057	−0.001
Model 2	175979.56	142085.91	6114	0.894	0.900	0.016 [0.016, 0.0016]	0.082	−0.009

*Model 0 = free model; Model 1 = Model 0 with factor loadings of first-order; Model 2 = Model 1 with factor loadings of second order; S-Bχ^2^ = Satorra-Bentler χ^2^ scaled; df = degrees of freedom; TLI = the Tucker-Lewis Index; R-CFI = robust comparative fit index; R-RMSEA = robust root mean square error of approximation; SRMR = standardized root mean square residual; ΔR-CFI = robust comparative fit index difference test.*

## Discussion

The present study examined the adjustment and reliability values of a 9-factor, 66-item measure of school climate and academic mindset among a very large sample of elementary, middle, and high school students. Confirmatory factor analysis supported the proposed factor structure of the School Climate and Academic Mindset Inventory (SCAMI) (i.e., Parent Involvement and Support, Academic Mindset, Social Emotional Learning, Safety, Physical Safety, Bullying, Physical Environment and Resources, Respect for Diversity, and Perceptions of School Performance). Coefficient values were adequate for all subscales (Taber, 2018). Model trimming to arrive at satisfactory goodness-of-fit indices included the removal of items from academic mindset (2 items), social emotional learning (11 items), and perceptions of school environment (2 items). Six other subscales retained their full item integrity and no items shifted from one factor to another. Removed items may constitute supplementary items to be used with caution. The subscale of Parent Involvement and Support demonstrated lower internal consistency and should also be used with caution.

In addition, the trimmed model displayed adequate goodness-of-fit for males and females, younger age groups, and European American, biracial/multiracial, Hispanic, Native American, and Native Hawaiian/Pacific Islander students. The trimmed model was slightly less strong for older age groups as well as for African American and Asian American students. School climate may be impacted by less social trust in later adolescence as well as differential discrimination among racial groups and percentage of minority students at a given school, which may have affected the results (Flanagan and Stout, 2010; Wang et al., 2014; Wang and Atwal, 2015). Factorial invariance analyses revealed that the SCAMI had an equivalent factor structure by gender and younger age groups. The constructs that the SCAMI is assessing can thus be viewed as comparable between males and females and individuals aged 9–11 and 12–14 years.

The assessment of school climate continues to evolve, and one advantage of the SCAMI is the inclusion of contextual variables utilized by students in their school-based endeavors, most notably academic mindset and social emotional learning factors. The scale allows researchers and school officials to more compactly study the relationship between various aspects of school climate and these associated variables, which is a burgeoning focus of the climate literature (Allbright et al., 2019). Researchers, for example, can use the scale in conjunction with grades and standardized test scores to better understand the specific mechanisms by which school climate leads to improved academic achievement (Cornell et al., 2016). In addition, educators and school-based mental health professionals could use the scale to identify schools, classrooms, and even individual students that may require additional support or services to enhance climate and, by extension, academic performance and well-being (Cleveland and Sink, 2017). The scale could be used to identify non-instructional targets of change in an academic environment (Zullig et al., 2010). The SCAMI could also be utilized as part of school climate improvement efforts within the context of multi-tiered systems of support models (James et al., 2018). In these models, Tier 1 system-wide strategies focus heavily on universal or primary prevention practices to promote adaptive behavior and deter maladaptive behavior, often via enhancement of positive school climate, adaptive mindsets, social-emotional competencies, well-managed and engaged classroom behavior, and successful academic performance (Voight and Nation, 2016). The SCAMI can be a practical tool for evaluating many aspects of Tier 1 intervention. In addition, the scale could help districts leverage limited resources by serving as a needs assessment to identify schools most in need of Tier 1 intervention to enhance school climate (Wang and Degol, 2016). This process could occur longitudinally as well to examine patterns of change over time or across grade and developmental levels (László et al., 2019).

Other tiers in a multi-tiered systems of support model include escalating interventions to address emerging (Tier 2) and later chronic and severe (Tier 3) academic, behavioral, social, and emotional problems (McIntosh and Goodman, 2016). The SCAMI may be utilized to help identify demarcations between these tiers so that schools have better benchmarks for shifting and intensifying the use of resources (Berkowitz, 2019). In addition, the scale may be helpful as part of an algorithm with other known benchmarks such as absenteeism, office discipline referrals, and course grades to help predict long-term outcomes such as school dropout (Kotok et al., 2016; Kearney et al., 2019).

Results from the present study also help confirm the substance of domains commonly ascribed to school climate. Dimensions of school climate are generally considered to be malleable in nature, with little agreement on specific labels among researchers except for wide-ranging relational and safety components (Grazia and Molinari, 2020). Several of the SCAMI school climate subscales can be broadly grouped into relational (parent involvement and support, respect for diversity, perceptions of school performance) and safety (physical safety, bullying) domains. Condition of school facilities, another common element of school climate assessment, is represented as well (Berman et al., 2018). A key advantage of the SCAMI is the presence of multiple items for each of these specific domains.

Limitations of the present study should be noted. First, the measure evaluated here (SCAMI) was derived from a prefabricated survey utilized by the school district involved in the present study. As such, the evaluation of the measure is more data-based than theory-based, and one primary geographical location was involved. In addition, less control was exercised over its distribution, with an unknown number completing the measure at home or school, which may have affected the internal consistency of some of the subscales. Second, the scale was based on student report despite the fact that school climate can affect youth, parents, and teachers, each of whom may evaluate climate differently (Ramsey et al., 2016). Student-based reports and inventories do have advantages, however, including practicality, compliance, and assessment of internalizing variables such as mindset (Yeager et al., 2016). Third, deeper student groupings, such as those with disabilities or mental health problems, were not feasible. Perceptions of school climate may differ among these student groups (La Salle et al., 2018). Finally, the scale was administered only in English; measuring school climate among Spanish-speaking and other-language students should be a future priority (Rocha et al., 2019).

## Conclusion

Despite these limitations, the evaluation of the SCAMI represents one of the largest and most diverse sample sizes with respect to school climate assessment. The results illustrated the adjustment and reliability values of the measure as well as factorial invariance across multiple demographic groups. Future work should focus on expansion of psychometric testing in other geographical regions, further validation, and multilevel modeling to explore individual and collective perceptions of school climate. In addition, the scale’s utility for assessing school climate interventions and longitudinal patterns, as well as its value within a multi-tiered systems of support model, should be fully evaluated. Finally, linking findings from the scale to other key school variables such as absenteeism, academic performance, social-emotional competency development, and relational aggression may be instructive.

## Data Availability Statement

The datasets generated for this study are available on request to the corresponding author.

## Ethics Statement

The studies involving human participants were reviewed and approved by UNLV IRB. Written informed consent from the participants’ legal guardian/next of kin was not required to participate in this study in accordance with the national legislation and the institutional requirements.

## Author Contributions

All authors revised and approved the submitted version. CK collected the data, wrote the manuscript, and supervised the study. RS and CG performed the analyses and assisted in writing of the manuscript.

## Conflict of Interest

The authors declare that the research was conducted in the absence of any commercial or financial relationships that could be construed as a potential conflict of interest.
